# Effectiveness and patient satisfaction with office hysteroscopic polypectomy in patients with symptomatic endometrial polyps

**DOI:** 10.52054/FVVO.14.4.045

**Published:** 2023-01-27

**Authors:** M.A. Céspedes Martínez, J Rovira Pampalona, M Degollada Bastos, R Izquierdo Argelich, J Bou Tapias, M.D. Flores Laura, P Brescó Torras, M.D. Carugno Jose

**Affiliations:** Department of Obstetrics and Gynecology, d’Igualada University Hospital, Barcelona, Spain; Obstetrics, Gynecology and Reproductive Sciences Department, Minimally Invasive Gynecology Division, University of Miami, Miller School of Medicine, Miami FL, USA

**Keywords:** Endometrial polyp, hysteroscopy, abnormal uterine bleeding, postmenopausal bleeding

## Abstract

**Background:**

Endometrial polyps are a common cause of abnormal uterine bleeding. In-office hysteroscopic management is frequently performed to treat this frequently encountered pathology.

**Objectives:**

To evaluate the long-term outcome and patients’ satisfaction with office hysteroscopic polypectomy in patients with symptomatic endometrial polyps.

**Materials and Methods:**

Retrospective longitudinal observational study of all hysteroscopic polypectomies performed at d’Igualada University Hospital (Barcelona, Spain) between May 2016 and December 2018. The medical records were reviewed, and a telephone interview was conducted with all the patients diagnosed with symptomatic endometrial polyps who underwent outpatient hysteroscopic polypectomy, with the purpose of evaluating the post-procedure symptomatology and satisfaction with the procedure.

**Main outcomes and results:**

A total of 848 outpatient hysteroscopies were performed, 379 of which were polypectomies. Of those, 163 procedures were performed in symptomatic patients and were included in the final analysis. The most common symptom among premenopausal patients was abnormal uterine bleeding (84.85%) and in postmenopausal women, postmenopausal bleeding (95.3%). After the procedure, the symptoms resolved or decreased considerably in 66.7% of premenopausal and 93.7% of postmenopausal patients. Additionally, 87.1% of the patients were very satisfied with the procedure.

**Conclusion:**

Office hysteroscopic polypectomy is an effective treatment for endometrial polyps with high patient satisfaction reported following the procedure.

## Introduction

Endometrial polyps are growths of the endometrial surface consisting of glands, stroma, and blood vessels, that proliferate independently in the surrounding endometrium (Nijkang et al., 2019). Its prevalence varies widely with a reported range between 7.8% - 34.9%, being more common in women between 40-50 years of age (de los Rios et al., 2015; Salim et al., 2011; AAGL, 2012). The aetiology is unknown, and various theories are postulated regarding the pathogenesis of these lesions. It is estimated that between 55% - 88% of endometrial polyps cause symptoms, this percentage being more frequent in premenopausal women (Salim et al., 2011; Vigueras et al., 2016). The most frequently reported symptom is abnormal uterine bleeding (AUB) in up to 68% of patients and is frequently described as intermenstrual bleeding in the premenopausal and as postmenopausal bleeding in menopausal women. Other reported clinical manifestations of endometrial polyps include infertility (32%) and pelvic pain (2%) (Stewart et al., 2022; Lieng et al., 2009).

The potential for malignancy inside endometrial polyps is reported to be up to 13% in patients with predisposing factors such as menopause, age over 60, diabetes, obesity, or the use of tamoxifen (Sasaki et al., 2018).

Currently, there is no consensus regarding the best strategy to treat endometrial polyps. Some promote hysteroscopic removal while others recommend expectant management with serial ultrasound evaluation, according to the symptoms reported by the patient, the risk factors for malignancy, as well as their association with infertility (SEGO, 2018).

Regarding treatment, hysteroscopy is considered the gold standard technique for the diagnosis and treatment of endometrial polyps. Office hysteroscopic polypectomy has proven to be a cost-effective procedure, with benefits for both the patient and the healthcare provider, with benefits including shorter recovery time, rapid incorporation into activities of daily living and a low complication rate (Marsh et al., 2006).

The objective of this study is to evaluate the outcomes of hysteroscopic polypectomy performed in our office hysteroscopy clinic in patients diagnosed with symptomatic endometrial polyps, as well as patients’ satisfaction with the procedure.

## Materials and Methods

A retrospective observational study was performed, including all patients who underwent outpatient hysteroscopic polypectomy between May 2016 and December 2018 at the d’Igualada University Hospital in Barcelona, Spain. The medical records were reviewed, and only those patients diagnosed with endometrial polyps who reported symptoms prior to the hysteroscopic polypectomy were included in the study. Patients who were asymptomatic at the time of the polypectomy and those who had a hysterectomy after the initial polypectomy or were using birth control pills were excluded from the analysis. All the patients included in the study were contacted for a telephone interview in February 2019, with a follow-up interval variation ranging 2-33 months.

The variables analysed were age at the time of the procedure, menopausal status, previous symptoms, procedure performed, the pathology result, and the occurrence of complications. The data was extracted from the medical record. Subsequently, the patients were contacted by telephone in February 2019 and asked about the current symptoms and the level of satisfaction with the hysteroscopic procedure.

The symptoms after the procedure were classified as follows:

- Absent: Total absence of symptoms.
- Decreased: The patient reported a subjective substantial improvement.
- No change: The patient reported that her symptoms were the same as before the hysteroscopic procedure.
- Unable to assess: The patient started to use medications (i.e., contraception) that modified the bleeding pattern shortly after the hysteroscopy.
- Recurrence: The patient reported the recurrence of symptoms after a brief period of improvement.

The level of satisfaction with the procedure was assessed using a likert-type scale from 0 to 10 with 0 being dissatisfied and 10 extremely satisfied. The patients were classified into 3 sub-categories: 0-3 not satisfied, 4-7 moderately satisfied, 8-10 very satisfied.

All the hysteroscopies were performed using the Olympus® 5 mm hysteroscope with a 5 Fr working channel for the insertion of semirigid graspers and/ or scissors as well as the bipolar energy system (VERSAPOINT®). In addition, the hysteroscopy with mechanical cutting energy (TRUCLEAR® 5.0 System) with a morcellation cannula was used in some patients. In all the procedures, the distension media used was normal saline with a fluid management system.

All hysteroscopies were performed by experienced hysteroscopists as well as by trainees (OBGYN residents of the third or fourth year) under direct supervision. The uterine cavity was entered using a vaginoscopic approach without the use of analgesia.

For statistical analysis, standard descriptive statistics were used to analyse the data. Categorical variables were reported as percentages. All statistical analyses were performed with SPSS version 26.0 (IBM Corporation, 2018).

## Results

During the study period, a total of 848 hysteroscopies were performed. Of these, pathology confirmed the presence of endometrial polyp in 379 patients. The mean age of the patients was 52.6 years (SD 12.33). Of those 185 (48.8%) were premenopausal and 194 (51.2%) post-menopausal.

**Figure 1:**
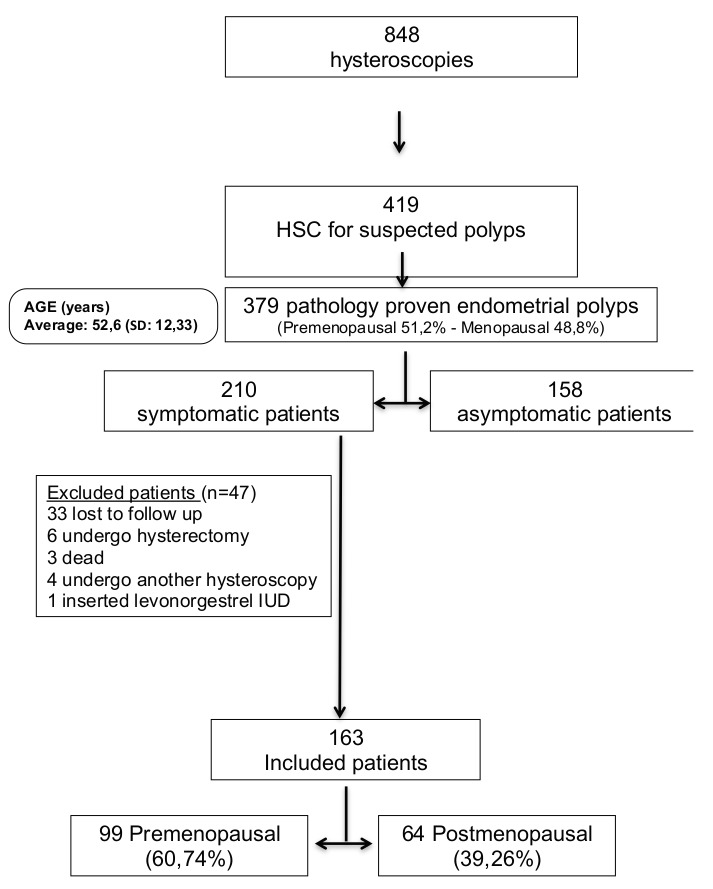
Summary table of the patients’ characteristics.

Of the patients undergoing hysteroscopic polypectomy, 41.6% (n=158) were asymptomatic and were excluded from the final analysis. A total of 210 patients (55.4%) reported symptoms, of these 47 were excluded from the analysis for various reasons. Of the 163 patients included in the final analysis, 99 were premenopausal (60.74%) and 64 were postmenopausal (39.26%) Figure 1.

In the premenopausal group, the most common symptoms were heavy regular menstrual periods (n=53). Among the post-menopausal patients, the symptom more commonly reported was postmenopausal bleeding (n=61).

At the time of the follow up phone interview, 49.5% of the premenopausal patients were asymptomatic. There were ten patients in whom it was not possible to assess their current symptoms due to being amenorrhoeic, either having reached menopause or through the use of a medicated intrauterine device. In the group of menopausal patients, most of the patients (90.6%) were asymptomatic. For the two remaining post-menopausal patients, the symptoms were unable to be assessed.

Regarding the degree of satisfaction with the procedure, the patients were asked to report on a scale of 0 to 10, with 0 being dissatisfied and 10 greatly satisfied with the experience of the office hysteroscopic polypectomy performed without anaesthesia. The majority of the patients (87.1%) were very satisfied and 12.9% were moderately satisfied. Of note, no procedure complications were reported.

## Discussion

Endometrial polyps cause symptoms such as AUB, infertility and pelvic pain in 55-88% of patients. Spotting in the form of unpredictable intermenstrual bleeding in the premenopausal, and as postmenopausal bleeding in the menopausal women is also frequently reported by patients with endometrial polyps (Salim et al., 2011; Stewart et al., 2022). Hysteroscopy is the gold standard for the diagnosis and treatment of endometrial pathology. Endometrial polyps have a reported spontaneous regression rate of 27% at 12 months, this probability of regression is higher in polyps smaller than 1cm and is lower in post-menopausal women (Lieng et al., 2009).

In our study, 55.4% of the polypectomies performed were in patients who were symptomatic at presentation. In our series, a non-negligible number of patients underwent outpatient polypectomy without presenting clinical symptoms. This practice is currently controversial, mainly in premenopausal patients (Ferrazi et al., 2009). On the other hand, the prevalence of malignancy in endometrial polyps should not be ignored according to the American Association of Gynecologic Laparoscopists (AAGL) position statement (AAGL, 2012), where the incidence was estimated between 0% - 12% depending on risk factors. Known risk factors for premalignant or malignant conditions in patients with endometrial polyps include advanced age, hypertension, obesity, and tamoxifen and/or hormone therapy use. Also, women affected by Lynch and Cowden syndrome have a higher incidence of endometrial pathology (Stewart et al., 2022; Lieng et al., 2009).

The greater risk of cancer in postmenopausal patients makes hysteroscopic polypectomy more frequently performed in this group of patients (Marsh et al., 2006). A more conservative management is accepted in asymptomatic premenopausal patients without risk factors for hyperplasia or endometrial cancer (Vigueras et al., 2016; Sasaki et al., 2018).

In our institution, hysteroscopic polypectomy is offered to all patients with ultrasound suspicion of endometrial polyp. The rationale for this practice is that hysteroscopy performed in an outpatient setting is considered a quick, inexpensive, and effective procedure, with a negligible complication rate.

The data obtained in our study indicates that 77% of our patients have improved symptoms after hysteroscopic polypectomy in the outpatient setting, with a very high level of patient satisfaction at 87.1%. Los Rios et al. reported similar results, showing the effectiveness of hysteroscopic polypectomy in terms of reducing AUB and patient satisfaction in a cross-sectional study, where 67.2% had symptom improvement and patient satisfaction of 82.8%.

In a systematic review published by Nathani and Clark (2006) ten observational studies were analysed, evaluating the efficacy of hysteroscopic polypectomy in the treatment of AUB. In all of them, symptom improvement rates were reported at 75-100%, with follow-up intervals ranging 2-52 months. Lieng et al. (2010) conducted a randomised control trial in 150 premenopausal women with the objective of estimating the clinical efficacy of hysteroscopic resection of endometrial polyps six months post-procedure. There were no significant differences in the volume of menstrual loss between the control group and the group in which the polypectomy was performed, however there was a significant decrease in the other symptoms, such as intermenstrual bleeding, in the patients who underwent hysteroscopic polypectomy.

Patient’s satisfaction with office hysteroscopic polypectomy was evaluated via telephone interview. In our study, the majority (87.1%) of the patients were very satisfied or satisfied with the procedure and the outcomes. The patients highly valued the speed and comfort of the outpatient setting, from the time of arrival to the discharge after the procedure, enabling them to resume their normal life soon after the procedure. In a randomised control trial (Marsh et al., 2006), patients who underwent office hysteroscopic polypectomy were compared to patients undergoing the same surgical procedure under general anaesthesia. The patients who had office hysteroscopy had less postoperative pain 24 hours after the procedure, less recovery time, and a quicker return to daily activities. There were no complications in either group (Marsh et al., 2004; Kremer et al., 2000).

In our hospital, in-office hysteroscopic polypectomies are performed without cervical preparation or the use of anaesthesia/analgesia. We counselled patients to take over the counter analgesic if desired after the procedure. A similar pain management approach was published by Kremer et al. (2000) with excellent patient satisfaction. Different methods have been proposed for pain control during hysteroscopies performed in an office setting, including oral analgesics, intracervical and paracervical block and up to intravenous conscious sedation (Luerti et al., 2019; Ahmad et al., 2010). However, the available evidence on pain control strategies during in-office hysteroscopy is controversial and their use is not yet routinely recommended (Del Valle et al., 2016).

In our study, there were no complications during the procedure. Office hysteroscopy is an effective procedure with a low complication rate (Aas-Eng et al., 2017). The literature reports between 0.4% - 4% of immediate and 0.001% long term complication rates (RCOG, 2011). It should be noted that these percentages differ between the type of procedure performed as well as the setting in which the procedure was performed (office setting versus operating room). In addition, the complication rate when the polypectomy is performed in an office setting is 0.4% (Luerti et al., 2019).

We must acknowledge that our study has several important limitations. First is the retrospective nature of the study. Second, the follow-up period in some patients has been of only a few months, which could be considered not enough time to evaluate the improvement of the symptoms in some patients. Also, we did not take into consideration the size, number, or location of the polyps. Third, asymptomatic patients were excluded from the final analysis which adds selection bias to the study and lastly, patient satisfaction with the procedure was evaluated a few months after the index procedure introducing recall bias.

However, we must also highlight some of the strengths of our study such as including cases performed by hysteroscopists with different levels of expertise, including residents in training.

## Conclusion

Hysteroscopic polypectomies performed in an office setting in patients with symptomatic endometrial polyps improves their symptoms with a high level of patient satisfaction following the procedure.
